# Neglected Agent Eminent Disease: Linking Human Helminthic Infection, Inflammation, and Malignancy

**DOI:** 10.3389/fcimb.2019.00402

**Published:** 2019-12-06

**Authors:** Naina Arora, Rimanpreet Kaur, Farhan Anjum, Shweta Tripathi, Amit Mishra, Rajiv Kumar, Amit Prasad

**Affiliations:** ^1^School of Basic Sciences, Indian Institute of Technology Mandi, Mandi, India; ^2^Cellular and Molecular Neurobiology Unit, Indian Institute of Technology Jodhpur, Karwar, India; ^3^Institute for Himalayan Bioresource Technology (CSIR), Palampur, India

**Keywords:** helminths, parasite, cancer, chronic inflammation, immune modulation

## Abstract

Helminthic parasitic infection is grossly prevalent across the globe and is considered a significant factor in human cancer occurrence induced by biological agents. Although only three helminths (*Schistosoma haematobium, Clonorchis sinensis*, and *Opisthorchis viverrini*) so far have been directly associated with carcinogenesis; there are evidence suggesting the involvement of other species too. Broadly, human helminthiasis can cause chronic inflammation, genetic instability, and host immune modulation by affecting inter- and intracellular communications, disruption of proliferation–anti-proliferation pathways, and stimulation of malignant stem cell progeny. These changes ultimately lead to tumor development through the secretion of soluble factors that interact with host cells. However, the detailed mechanisms by which helminths introduce and promote malignant transformation of host cells are still not clear. Here, we reviewed the current understanding of immune-pathogenesis of helminth parasites, which have been associated with carcinogenesis, and how these infections initiate carcinogenesis in the host.

## Introduction

According to National Cancer Institute, USA, cancer is “a term for disease in which abnormal cells undergo uncontrolled division and invade nearby tissues (metastasis).” In the year 2018, the World Health Organization (WHO) accounted 18.1 million new cases and 9.6 million deaths due to cancer (Ferlay et al., 2019). Arising from a single cell, progression of cancer has a very complex mechanism involving interaction between a number of factors (like age, sex, nutrition of host, exposure to pollutants, etc.), including host's genetics and carcinogens. Carcinogens are broadly classified as physical, chemical, and biological. Distribution of various types of cancer and incidence of associated mortality varies globally; however, the most common type of cancers responsible for mortality are lung, liver, stomach, colorectal, breast, and esophagus cancers (Brindley and Loukas, 2017; Ferlay et al., 2019). Among these, liver, stomach, colorectal, and esophageal cancers are often found to be associated with distinct infectious diseases or biological agents (WHO, 2015). The International Agency for Research on Cancer (IARC) had classified 11 species of pathogens as group 1 carcinogen, which means “definitely carcinogenic to humans” (IARC, 2012). The biological carcinogens include viruses (hepatitis B virus, hepatitis C virus, human Papilloma virus, Epstein–Barr virus, human T-cell lymphotropic virus type 1, human herpes virus type 8, and human immunodeficiency virus type 1), bacteria (*Helicobacter pylori*) and helminths (*Opisthorchis viverrini, Clonorchis sinensis*, and *Schistosoma haematobium*), which collectively account for 17.8% of cancer cases (de Martel et al., 2012; IARC, 2012).

Although incidence of helminth-induced cancer is relatively very low, but widespread prevalence of helminth infections across the globe collectively predisposes large number of populations to cancer risk. According to Global Disease Burden estimates, nearly more than one billion people living in underprivileged regions of sub-Saharan Africa, Asia, and America are exposed to either one or more helminthic species every year (WHO, 2012; Global Burden of Disease Study 2013 Collaborators, 2015). However, the reported incidence of helminth-induced cancer is very low. One of the probable reasons could be that these parasitic infections are prevalent among low-income population in third world countries, which have poor medical facility. This makes proper identification of etiological agent of cancer cumbersome. Infection with liver flukes *O. viverrini* and *C. sinensis* can induce cholangiocarcinoma (CCA), and infection with blood fluke *S. haematobium* causes cancer related with urinary bladder (Bouvard et al., 2009). Other parasites are also reported to be associated with malignancy; however, their association is yet to be firmly established. In a recently published meta-analysis, authors reported 12 parasitic infections (seven helminths and five protozoans) in patients with cancer or tumor (Machicado and Marcos, 2016). In this study, the helminths identified to be associated with cancer were *Fasciola gigantica, Taenia solium, Echinococcus granulosus, Strongyloides stercoralis, Trichuris muris, Platynosomum fastosum*, and *Heterakis*, and the protozoans identified were *Theileria, Cryptosporidium parvum, Trichomonas vaginalis, Plasmodium falciparum* and *Toxoplasma gondii*. Although, Plasmodium is not categorized as carcinogen by IARC it is well-documented that the malaria parasite acts as a co-carcinogenic factor for the development of Burkitt lymphoma with Epstein–Barr virus coinfection (Molyneux et al., 2012). It is difficult to decipher helminth's contribution in cancer development due to its long period of latent infection and time taken to develop malignancy. The host may have encountered numerous infections and coinfections of one or more infectious agents before the first appearance of cancer symptoms. By the time first symptoms of cancer appear, no eggs or signs of helminthic infection might remain to specifically associate species of helminths to cancer. Moreover, the complex asymptomatic phase of helminthic infection adds more complexity. The list of helminthic species and the type of cancer they induce is listed in Table 1. Besides these limitations, helminthic infections fall in the category of tropical and neglected diseases; hence, very few efforts have been performed to understand their immuno-pathogenesis. Fortunately, now, the scientific community is putting more effort to understand the helminths biology and their immuno-modulating and carcinogenic properties (Prasad A. et al., 2009; Sripa et al., 2009; Botelho et al., 2012; Vale et al., 2013). In this review, we analyzed existing literature related to association between helminths and malignancy and discuss some possible mechanisms of helminth-induced carcinogenesis.

**Table 1:** Helminthic parasite, their associated disease, mechanisms of carcinogenesis, and associated cancer.

**Parasitic pathogen**	**Endemic areas**	**Disease**	**Suggested mechanisms of carcinogenesis**	**Associated cancer**	**References**
*Opisthorchis viverrini*	Southeast Asia	Opisthorchiasis	Inflammation, oxidative stress caused by parasite-derived molecules, cell proliferation, *H. pylori* mediated induction	Cholangiocarcinoma	Pinlaor et al., 2004; Ninlawan et al., 2010; Khuntikeo et al., 2016; Pakharukova and Mordvinov, 2016
*Opisthorchis felineus*	Eastern Europe, Southeast Asia	Opisthorchiasis	Inflammation, oxidative stress caused by parasite-derived molecules, cell proliferation	Cholangiocarcinoma	Maksimova et al., 2015; Pakharukova and Mordvinov, 2016
*Clonorchis sinensis*	China, Korea, northern Vietnam	Clonorchiasis	Inflammation, oxidative stress caused by parasite-derived molecules, cell proliferation	Cholangiocarcinoma	Lim et al., 2006
*Schistosoma haematobium*	Africa, Middle East	Schistosomiasis	Inflammation, oxidative stress caused by parasite-derived molecules	Urinary bladder cancer, adenocarcinoma, squamous cell carcinoma	McCormick et al., 1996; Mostafa et al., 1999; Botelho et al., 2013; Machicado and Marcos, 2016
*Schistosoma japonicum*	Asia	Schistosomiasis	Inflammation, oxidative stress caused by parasite-derived molecules	Colorectal cancer, rectal cancer, squamous cell carcinoma, membranous nephropathy, metastatic lung cancer	Takemura et al., 1998; Zhang et al., 1998; Zanger et al., 2010
*Schistosoma mansoni*	Central America, South America, sub-Saharan Africa	Schistosomiasis	Inflammation, oxidative stress caused by parasite-derived molecules	Adenocarcinoma, colorectal cancer, hepatocellular carcinoma	Barral-Netto et al., 1983; Zaghloul and Gouda, 2012

## Effect of Chronic Inflammation and Immune Evasion Strategies

Parasitic infections and the subsequent host immune response are outcomes of prolonged dynamic coevolution between the host and parasite. For parasites, it is a necessity to trick the host defense system from developing an effective immune response for their survival (Colley, 2000). Severe changes in phenotype of hosts infected with parasite are often noticed particularly in parasites that have intermediate host. However, how these changes make host/parasite suitable for survival is still not clear (Cézilly et al., 2010). Extracellular parasites are very smart and can evade immune system detection for a very prolonged period by immune modulation of host; they acquire these lifesaving skills due to constant exposure to hostile environment and threat from host immune system. The host mechanisms of defense against multicellular parasites range from simple primary epithelial cell barriers to the most elaborate signaling mechanisms, involving different kind of immune reactive cells and molecules. All immune dodging machineries are extremely embedded in the fine details of the cellular and molecular mechanisms that regulate the immune responses. These parasites had evolved immune evasion strategies that depend upon numerous factors like synthesis and secretion of immune evading molecules, life cycle, stage, route of infection, and the tissue microenvironment in which they multiply or survive in host (Sripa et al., 2009; Botelho et al., 2012; Vale et al., 2013; Brindley et al., 2015). These constant hostile environments for parasites push them for host switching, which involves several stages like opportunity (for host switching), compatibility (for long-term association), and conflict resolution (for living together) (Araujo et al., 2015). A parasite constantly keeps developing various self-defense factors, which may be due to lack of conflict resolution mechanism may become detrimental for host.

**Figure 1:**
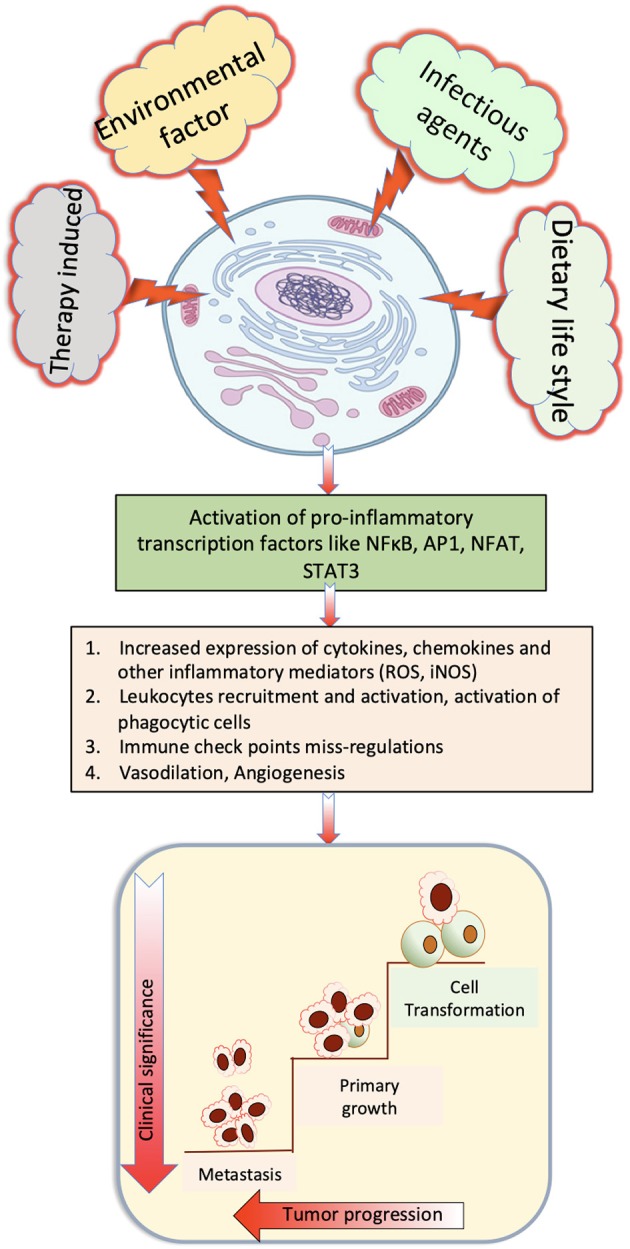
Role of various stimulatory signals that cause chronic inflammation that finally induce cancer.

Parasite-derived factors are involved in initiation, promotion, and progression of tumor. The precise nature of host immune response against pathogens varies noticeably between different species. However, a T helper 2 (Th2) phenotype is a conserved immune response to helminthic infection in mice and humans and is marked by the production of significant amount of Programmed death-1 (PD-1), IL-4, and IL-10 (Maizels, 2009; Singh et al., 2013; Zhou et al., 2016; Assunção et al., 2017). Inflammatory cells produce reactive oxygen species (ROS) and reactive nitrogen species that, apart from neutralizing bacterial infection, can oxidize genetic material and damage DNA or induce frame shift mutations or chromosomal abnormalities in the cells, and these are one of the main elements that induce cancer (Parascandolo and Laukkanen, 2018). Excretory secretory products of parasites are immunomodulatory, and increased PD1 expression reduces T-cell activation with the help of co-stimulatory molecules PDL1 and PDL2. Alternatively activated macrophages that suppress Th1 and promote Th2 immune response are prominent in chronic parasitic infections that express high PD-L1 and PD-L2, thus promoting T-cell tolerance (Stempin et al., 2016), which also helps in promotion of cancer. Prolonged Th2 immune response leads to chronic stress and downregulated immune response, which promotes chronic inflammation that had been reported to be associated with occurrence of cancer (Jovanovic et al., 2014; Partecke et al., 2016). Various stimulatory signals and important cellular pathways that cause chronic inflammation and steps to progression of cancer are depicted in Figure 1.

## Mechanisms of Carcinogenesis

The loss of gene function of a tumor suppressor gene resulting in oncogene or proto-oncogene onsets the uncontrolled proliferation of cells that causes cancer. There are number of factors/mutagens that contribute to loss of regulated cell growth. The stages of cancer development can be classified as: initiation, promotion, and progression. Initiation begins with the alteration causing damage to DNA irreversibly by either xenotoxic carcinogens/mutants or chronic inflammation (Botelho et al., 2012; Brindley et al., 2015). The damage can be caused by host of xenobiotic compounds. Once the gene has been mutated, it loses its control over function and the homeostasis. Over the period, mutation accumulates in the genes, and there is rapid proliferation of cells resulting in neoplastic growth. Several types of cancer may arise from the site of infection having chronic inflammation. Chronic inflammation caused by parasites or depositions of parasite products or antigens in tissues are the key features in helminth-induced carcinogenesis (Brindley and Loukas, 2017). Chronic inflammations in response to parasitic infection or in response to parasite-derived factors are associated with significantly elevated IL-6 and marked increase in fibrosis (Jovanovic et al., 2014). Initiation of tumorigenesis is promoted by reactive ions (ROS/reactive nitrogen species) made in response to oxidative stress due to parasitic products. Synthesis of TNF-α and TGF-β helps in autonomous proliferation, evasion of apoptosis, and angiogenesis of the incipient neoplasm (Sripa and Kaewkes, 2002). The tumor microenvironment generated by inflammatory cells during chronic inflammation is a crucial element in the neoplastic process. The tumor changes the microenvironment to facilitate its growth, and depending upon the nature, it may be benign or malignant. The malignant tumors spread to other parts of the host by traveling through the blood vessels.

**Figure 2:**
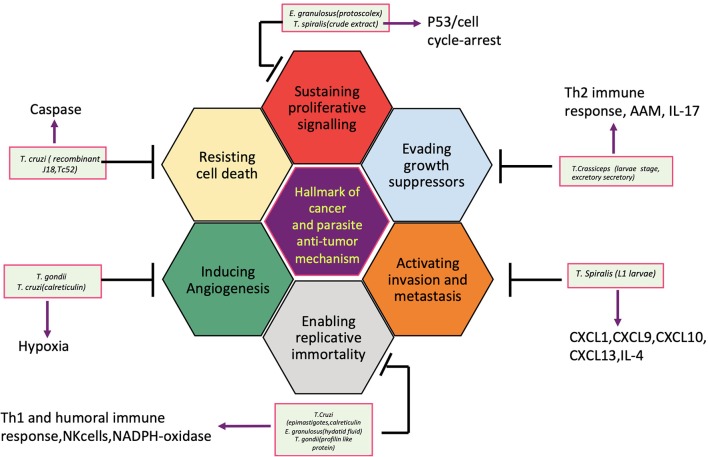
Hallmark of cancers and the helminth parasites antitumor mechanisms.

**Figure 3:**
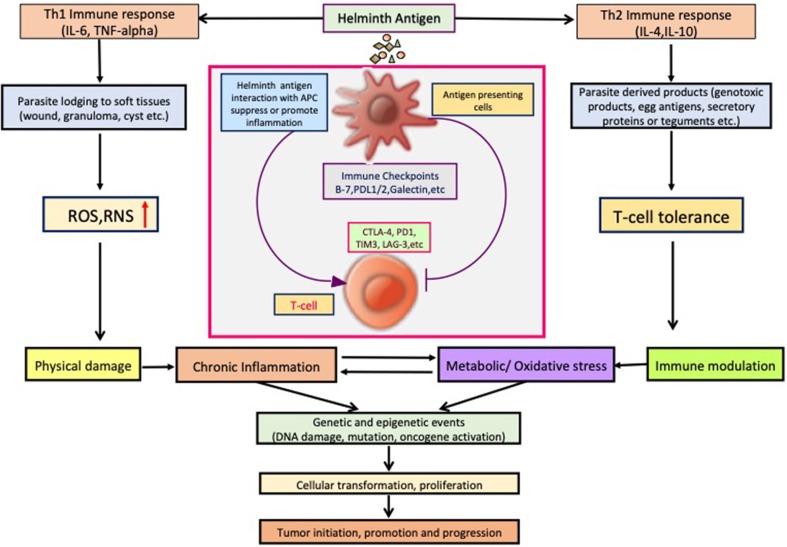
Helminth infection leads to inflammation, immune modulation, physical damage, and metabolic stress in host cells and disturb key cellular processes regulating cell proliferation, hereby inducing carcinogenesis.

So far, several parasites, helminths specially, have been associated with the etiology of human cancer; however, the knowledge of the mechanisms or pathways by which these parasites induce malignant transformation of their host cells is unclear. Figure 2 displays how helminth infection leads to inflammation, immune modulation, and metabolic stress that affects host cell and disturbs key genetic and epigenetic processes regulating cell proliferation, hereby inducing carcinogenesis in infected individuals. Inflammatory cells such as neutrophils, macrophages, and eosinophils generate good amounts of free radicals and nitrogen species in response to the parasites/bacteria. These reactive ion products can oxidize and damage DNA or initiate point mutations in DNA that may cause genetic instabilities and consequently malignant transformation of affected cells. Repeated tissue damage caused by the parasites or their eggs or secreted products leads to restorative hyperplasia of the damaged tissue (Sripa et al., 2009; Botelho et al., 2012; Vale et al., 2013; Correia da Costa et al., 2014; Brindley et al., 2015; Brindley and Loukas, 2017). After this stage, it is a matter of time and further genotoxic events before a potential cancer incidence. Helminths affect all the hallmarks of cancer, and it has been depicted in Figure 3.

In helminthiasis, the chronic infections and heightened inflammation in the infected tissue lead to neoplasma formation and associated pathology. Similarly, schistosome eggs entrapped in the bladder wall release various metabolites that induce chronic inflammation, finally leading to urothelial hyperplasia, dysplasia, and squamous cell cancer (SCC). In an experimental model of secondary echinococcosis and breast cancer, mice inoculated with protoscolexes and breast tumor cells had increased CD4+ T cells in the liver and spleen and reduced IFN-γ+ and CD25+ T-cells (Botelho et al., 2009b, 2013). They also observed increased frequency of metastasis in the liver, suggesting an immunological link between cystic echinococcosis and cancer that allows tumor metastasis to flourish in the liver. In our previous study regarding cytokine response among symptomatic and asymptomatic neurocysticercosis (NCC) on peripheral blood mononuclear cells, we have observed predominant Th2 response (increased IL-4 and IL-10) in asymptomatic cases (Prasad A. et al., 2009). In a swine study, we observed that Th2 and regulatory cytokine responses were associated with cysticerci non-responder to albendazole treatment and hence prolonged inflammation (Prasad K. N. et al., 2009; Singh et al., 2015), suggesting a possibility of oncogenesis and cysticercosis. However, so far, no report is available where prevalence of malignancy in asymptomatic NCC cases was investigated, except few reports where *Taenia* infection had been correlated with malignancy (Herrera et al., 2000).

According to an IARC monograph, so far, only three helminths have been classified as definitely carcinogenic (group 1 carcinogens), namely, *S. haematobium, C. sinensis*, and *O. viverrini*; therefore, only these three helminths are discussed in detail in this review. Few helminths whose association is not so well-defined are also included in this review for better understanding of the current scenario and future perspective.

## Trematode

Flukes contribute majorly for overall disease burden among the neglected tropical diseases, especially digenetic trematodes such as *Schistosoma* spp., *Fasicola* spp., *C. sinensis, Paragonimus* spp., *Eurytrema pancreaticum, Fasciolopsis buski, Philophthalmus lacrimosus*, etc. Depending on the site of infection, it may be categorized as either a tissue fluke or a blood fluke. These flukes need a mollusk (intermediate host) and a vertebrate (primary host) to complete their life cycle. Their prevalence is scattered across the continent, and *Schistosoma* is most important species among them.

## *Schistosoma* spp.

Blood flukes (*S. japonicum, S. haematobium*, or *S. mansoni*) cause schistosomiasis, which is also known as bilharzia (Barral-Netto et al., 1983; Vos et al., 2013; Colley et al., 2014). This affects nearly 230 million people worldwide and is known to be the second most common parasitic infection of human (WHO Expert Committee, 2002). Schistosomes are the only bisexual organisms of the trematode family. Their life cycle contains both asexual and sexual phases. The male and female parasites invade the host vascular system and produce eggs that are either excreted or trapped by nearby tissues. The eggs that get entrapped in nearby tissues form granulomatous structure with parasite part/products entrapped, keep stimulating host immune system to generate inflammatory response. The egg antigens induce alternatively activated macrophages along with IL-4 and IL-5. Soluble egg antigens (sh-SEA) of the parasite have also been found to have tumorigenic properties. In an *in vitro* study, it was noticed that sh-SEA can downregulate estrogen receptors (ER) alpha and beta in estrogen-responsive cells. It was noticed that sh-SEA at even very low concentration (6.25 μg/ml) can induce cell proliferation, induce oxidative stress, and reduce apoptosis in urothelial cells (Botelho et al., 2013). Schistosomes also make an estradiol-like molecule that can suppress ER leading to suppressed transcriptional activity of ER in HCV29 cell lines and bladder of schistosome-infected mice (Botelho et al., 2012). The proteolytic enzymes and metabolites are released by eggs that help their egress to urine. However, this also leads to hematuria and chronic inflammation of bladder wall. In a study from Angola, estrogen-like metabolites (catechol estrogen quinones and catechol estrogen quinone-DNA-adducts) were identified in 7/40 urine samples of patients with urogenital sinus (Gouveia et al., 2015). These metabolites are known for their carcinogenic properties. The chronic inflammation marks the onset of the disease schistosomiasis, which increases risk of urothelial hyperplasia, dysplasia, and SCC (Parkin, 2006; Wishahi et al., 2014). The infection can be acute, with symptoms like headache, fever, fatigue, abdominal pain, etc. The chronic symptoms differ with intensity of infection and can vary from intermittent abdominal pain to rectal bleeding, etc. The poorly regulated infection can lead to liver fibrosis and urogenital schistosomiasis that may develop into schistosomiasis-associated bladder cancer (SABC).

Although *S. mansoni* endemic zones are widespread across several continents (Africa, Eastern Brazil, and South America), they commonly coinfect with *S. haematobium* in several places. However, no definitive reports are available to link their infection with the geographical occurrence of cancer. For another trematode, *S. japonicum*, there are some evidences though not definitive, which suggests its direct association in the induction of liver cancer in Japan and colorectal cancer in China (World Health Organization, 1994). Several studies from these two countries have suggested a causal link between schistosomiasis caused by *S. japonicum* and more common gastrointestinal malignancies such as colorectal carcinoma, hepatocellular carcinoma, and/or colorectal cancer (Zanger et al., 2010). Pathological records, analysis of hospital records, and tissue archives have shown a correlation between this infection and cancer, and several case reports also suggested their direct association. In a study from China, among patients with rectal cancer, very high p53 tumor suppressor gene mutation was found in patients with schistosomal-associated rectal cancer, although this was statistically not significant (*P* = 0.054) (Zhang et al., 1998). Epidemiological analyses conducted in China and Japan support the role of *S. japonicum* infection as one of the risks factors in cancer formation, along with other cofactors such as hepatitis virus and alcohol intake but not as sole etiological agent (Takemura et al., 1998). Several animal experiments have also demonstrated that cancer develops early and in higher numbers in experimentally infected *S. japonicum* animals when any known carcinogen is administered to the same animal. However, in spite of all these positive end point associations, the mechanism of schistosome-mediated augmentation of carcinogenesis is unclear and illusive. Therefore, the only trematode that is known and accepted as a carcinogen is *S. haematobium*. This parasite is extensively studied for its association with bladder cancer; hence, it is discussed in detail in this review.

## Epidemiological Evidences

The association of occurrence of bladder cancer with *Schistosoma* infections seems to be deeply related to the endemicity of the parasite. The unanimity of available knowledge strongly indicates an association between *S. haematobium* infection and the incidence of bladder cancer. Ferguson (1911) was the first person to associate schistosomiasis and bladder cancer in Egypt in early twentieth century. In countries with high prevalence of *S. haematobium* like sub-Saharan Africa, Egypt, Iraq, Zambia, Malawi, and Kuwait, bladder cancer is the most common type of cancer, and SCC is the most frequent histological type in male and second most common finding in female (Groeneveld et al., 1996; Mostafa et al., 1999). In contrast, bladder cancer is the 5th or 7th most common cancer in males and 7th or 14th in women, in countries free of *Schistosoma* like United States, Germany, or United Kingdom (Sanli et al., 2017). In a meta-analysis of cross-sectional studies from sub-Saharan Africa, 70/682 million individuals estimated to have hematuria in the past 2 weeks that authors found were directly associated with *S. haematobium* infection (van der Werf et al., 2003). In case of *S. haematobium*, eggs are primarily deposited together in the form of a large conglomerate in the bladder wall, and this causes ulceration and hematuria, and eggs are released in the urine. *S. haematobium* eggs are present in up to 33% of women in endemic areas (Leutscher et al., 2000; Poggensee et al., 2000; Kjetland et al., 2005; Santos et al., 2014). The significant association of catechol-estrogens/DNA adducts and infertility was linked with ovarian hormonal imbalance due to *Schistosoma* infection (Santos et al., 2014). Mostafa et al. (1999) noted that rural school-going children are at higher risk of infection due to their proximity to infected water. Case–control studies looking for association between the occurrence of urinary bladder cancer and infection with *S. haematobium* have shown significant positive associations, with estimated risks ranging from 2 to 14 (Vizcaino et al., 1994; Bedwani et al., 1998). Eggs may also get deposited in the genital organs in both males and females and causes cervical lesions. Eggs retained in the urinary bladder wall can induce inflammation and thickening of the bladder wall and pseudopolyps. Later on at chronic stage, bladder wall masses may start developing fibrosis and calcifications, and secondary bacterial infection is a common observation in SABC (Gryseels et al., 2006).

## Histopathological and Experimental Evidences

The histopathological entities of SABCs have certain distinct features that differ from those of bladder cancer found in Western countries; they are of low or moderate grade, lymph node metastasis is higher, and their prognosis is also better compared with non-bilharzial bladder (Shokeir, 2004). The presentation of SABC is frequent with symptoms of cystitis, painful micturition frequency, and hematuria (>70%). Urography usually reveals an extensive irregular filling defect in the cystographic phase. In a cross-sectional study from *S. haematobium*-endemic Msambweni area of Kenya, urine cytology findings among 1,014 residents showed that prevalence of inflammation (39%), hyperkeratosis (30%), metaplasia (33%), and frank atypia (0.4%) were remarkably higher than those in other non-endemic populations (Hodder et al., 2000). It was also reported that infection of *S. haematobium* was strongly associated with increased risk of cytologic abnormality (2.8-fold relative risk of metaplasia or hyperkeratosis; *P* = 0.001). This parasite has been found to be involved in altering the expression of xenobiotic- and carcinogen-metabolizing enzymes too. In a study from Egypt, free radical level increased significantly (up to 57%) in infected bladder cancer tissues but not in uninfected tissues (Sheweita et al., 2004). They also noticed reduced glutathione-s-transferase and N-nitrosodimethylamine-N-demethylase I activity while increased aryl hydrocarbon hydroxylase activity in SABC.

Different animal models have been studied to understand the role of *S. haematobium* in inducing SABC. Experimentally infected talapoin monkey (*Cercopithecus talapoin*), capuchin monkey (*Cebus appella*), gibbons (*Hylobates lar*), opossums (*Didelphys marsupialis*), etc. have been used for this purpose. In all these models, SCC was the end result (Obuyu, 1972; Cheever et al., 1974; Hicks et al., 1980). These types of carcinoma were morphologically similar to those observed in human bladder (Zaghloul and Gouda, 2012), and this further confirms that there is a definite association between *S. haematobium* and bladder cancer. Development of animal models had helped immensely in understanding of the disease. Fu et al. (2012) developed new mice model by microinjection of purified *S. haematobium* eggs to bladder wall of mice. These mice consistently developed macrophage-rich granuloma and passed egg consistently for next 3 months. Chala et al. (2017) injected *Schistosoma* eggs into bladder of mouse to investigate urinary bladder cancer, and they found squamous metaplasia in eggs + N-nitrosodimethylamine group at 12 weeks but not in any other group and thus introduced a new promising tool to study *S. haematobium* egg-induced urinary bladder cancer. Immunopathological role of *S. haematobium* antigens is also well-established in inducing cancer via cell cycle control or gene deletion, methylation, and/or mutation in tumor suppressor genes or oncogenes (Warren et al., 1995; Eissa et al., 2000). Several key molecules related with immune activation/suppression had been identified for this infection. The role of PD-1 is also reported in *Schistosoma* infection. Activation of PD-1 signaling is crucial for CD4+ T cell function. Zhou et al. (2016) found increased expression of PD-1 in CD4+ T cells of infected patients and in spleen, mesenteric lymph nodes, and liver of mice with *S. japonicum* infection. Blockade of the same resulted in severe liver pathology, suggesting enhanced Th2 cell activity (Zhou et al., 2016). At the same time, this immune modulation by parasite toward Th1 response increases their survival chance. Infection of *Schistosoma* has been associated with loss of function to p15, p16, and p27 genes due to gene deletion, which are known as tumor suppressor genes; they encode proteins to negatively regulate G(1)-S cell cycle check point (Botelho et al., 2009b; Eissa et al., 2015). In an Egyptian study involving 168 tumor tissues, all the SABCs and SCC cases were having this deletion (Eissa et al., 2000). This observation was further confirmed in a mice model study (Botelho et al., 2009a), when CHO cells treated with *S. haematobium* total protein were injected in nude mice; all the mice developed cancer and vimentin filaments and were negative for cytokeratin. Another important metabolic enzyme cytochrome P450 (CYP) was also found to be associated with *Schistosoma* infection outcome (Cardoso et al., 2017). In an African population-based study, 28.5% of *Schistosoma-*infected patients were carriers of CYP2D6 ^*^5/^*^5 and IL6-174C polymorphisms, and it was associated with increased severity and morbidity of disease, thus confirming their role in cancer induction.

## Suggested Mechanisms of *S. haematobium* Induced Carcinogenesis

Numerous mechanisms have been described or suggested for induction of cancer by *Schistosoma*. However, at present, the available information suggests that this is a multifactorial multistage process that involves several stimulants, chronic inflammation being the center of all these mechanisms. The whole genome sequence of *S. haematobium* was published in late 2012, which showed 92% sequence identity with *S. mansoni* (Young et al., 2012). *S. haematobium* egg deposition in the urinary bladder induces chronic inflammation, irritation, and accumulation of genotoxic products. All these events are associated with increased genomic instability and host cell proliferation thus increasing risk of cancer initiation at the site of inflammation. These mechanisms had been identified by studies done on human tissue samples. The cell cycle regulatory protein p53 was first to be evaluated for its role in SABCs. In a study from Egypt, 92 patients with carcinoma were studied, and base pair substitution in exon 5–8 of the p53 gene was found significantly higher (*P* = 0.003) in SABC (Warren et al., 1995). The role of DNA repair/damaging enzymes was also analyzed. An analysis of samples of bladder tissue and bladder cancer from patients infected with *S. haematobium* revealed high levels of pro-mutagenic DNA lesions such as O6-methyldeoxyguanosine (Badawi et al., 1994). Moreover, bladder tissue is known to have diminished DNA repair capacity, including decreased O6-alkylguanine-DNA-alkyl-transferase activity. In order to analyze the role of CpG methylation in initiation of SABCs among Egyptian population, 41 samples of cancerous bladder tissue were tested for methylation in 12 cancer-associated genes using polymerase chain reaction. All but two cases had at least one methylated gene; 45% had three or more methylated genes, thus confirming increased epigenetic changes in the urothelium of SABCs (Gutiérrez et al., 2004). This observation confirmed the role of gene methylation in initiation and progression of SABCs. Later studies identified various other molecules/genes involved in cell cycle control or mutations in tumor-suppressor genes/oncogenes associated with occurrence of SABC (Tamimi et al., 1996; Eissa et al., 2000). The role of ROS molecules is also well-established in SABC. Initially, it was reported that in mouse model of *Schistosoma*, eosinophil cells isolated from experimentally induced granuloma were unable to reduce ferricytochrome-C following propidium monoazide treatment, which suggests increased expression of free radicals in these cells (McCormick et al., 1996). Later, increased expression of inducible nitric oxide synthase (iNOS) and DNA repair enzyme 8-hydroxy-2'-deoxyguanosine was noticed in *Schistosoma-*infected patients with urothelial carcinoma (Salim et al., 2008). These molecular and cellular changes associated with inflammation make the surrounding cells/tissues prone to subsequent oncogenic stimulation. It has been noticed that on administration of liver carcinogen 2-amino-5-azotoluene in mice, the risk of developing hepatic carcinoma increased in the presence of *Schistosoma* infection. Similarly, epithelial hyperplasia and metaplasia were found in the bladders of mice that had been infected with *S. haematobium* after pretreatment with an aromatic amine such as acetylaminofluorene (Hicks et al., 1980) or following a mechanically induced *Escherichia coli* infection in combination with 2-naphthylamine treatment. All the previously discussed findings suggest the role of *Schistosoma* in increased susceptibility of host to carcinoma.

## Liver Flukes

The fish-borne trematodes such as *O. viverrini, Opisthorchis felineus*, and *C. sinensis* are the most widespread liver flukes imposing risk of infection to ~700 million people worldwide (Keiser and Utzinger, 2009). The highest incidence of liver fluke infections is associated with CCA especially in regions of Eastern Europe, Southeast Asia, China, Thailand, Laos PDR, Vietnam, and Cambodia. The life cycle is similar for both *Opisthorchis* and *Clonorchis* species, with snail being the first intermediate host and fish the second intermediate host. The eggs that are ingested by snails (Hydrobiidae, Bithyniidae, and Malaniidae families) go through various developmental stages and release cercariae, which penetrate the second intermediate host, i.e., freshwater fish. More than 130 species of fish can be the second intermediate host, but three most commonly involved species are *Cyclocheilichthys, Puntius*, and *Hampala dispa*. The cercariae encyst underneath the fish skin in muscles and form metacercariae. After ingestion of infected undercooked or raw fish, the metaceracariae excyst in the duodenum and then ascend to biliary duct via hepatopancreatic duct and mature into adult worms over a period of a month. The adult worms attach to the mucosa, and host remains asymptomatic for a prolonged period. The disease outcome depends on the parasitic load, location and duration of infection along with host's immune-incompetence (Kaewpitoon et al., 2008). Adult liver fluke usually resides in medium-sized or small intra-hepatic bile ducts of the human biliary tract. This intraductal localization of flukes causes mechanical obstruction, inflammation, adenomatous hyperplasia, and periductal fibrosis or intrahepatic liver cancer. Pathophysiology and clinical manifestations of these two liver flukes are very similar (Lun et al., 2005; Marcos et al., 2008).

## Epidemiological Evidences

*C. sinensis, O. felineus*, and *O. viverrini* are major causative agents of bile duct cancer (CCA) in their endemic areas. The first report of *O. viverrini* infection came from Thailand in 1915 (Leiper, 1915). Later on, it became endemic to Southeast Asian countries, including Laos, Cambodia, Thailand, Vietnam, and with some sporadic incidences in Malaysia, Singapore, and Philippines (Sripa et al., 2010). In a case report involving autopsy of human infected with the parasite in 1953, it was found to be associated with bile duct and liver cancer (Viranuvatti and Mettitawongse, 1953). In an analysis of data from the National Cancer Registry of Thailand, the proportionate incidence of CCA (3.1) and hepatocellular carcinoma (1.2) was highest in Khon Kaen province in Northeastern Thailand, which coincided with the highest prevalence of *O. viverrini* infection (Srivatanakul et al., 1988). The prevalence of CCA in infected patients was unusually higher compared with that in people living outside the endemic area in Thailand, and hence, this parasite is considered as an etiological agent for cancer. In another study from the same area, fecal egg count was measured for *O. viverrini* and compared with the National Cancer Registry of Thailand. The authors found positive association between parasite infection and occurrence of CCA (Sriamporn et al., 2004). In 1874, more than 140 years ago, *C. sinensis* was first found in the bile duct of a young Chinese man during autopsy in Calcutta, India (McConnell, 1875). At present this parasite infects annually more than 35 million people in countries like Korea, Taiwan, Vietnam, Russia, and almost half of the population of China (Keiser and Utzinger, 2009). In an initial study, 87 adults from a small village of northeast Thailand were screened, and six out of eight adults infected with liver fluke infection were confirmed for CCA by computerized tomography (CT) scan (Elkins et al., 1990). Similarly, in another study from Thailand, enlargement of liver was examined by ultrasonography among residents of 24 highly endemic localities. In a hospital-based study from Korea, 3,080 consecutive patients with gastrointestinal symptoms were studied for prevalence of clonorchiasis. CCA was significantly higher (*P* = 0.008) in *C. sinesis*-infected vs. non-infected patients (Kim et al., 2009), suggesting etiological role of *C. sinesis* in CCA. Among infected adults' indistinct gallbladder wall, gallbladder sludge and enhanced portal vein radicle echoes were most frequent observations, highlighting the importance of infection and its association with frequency and severity of fluke-related hepatobiliary clinical condition (Mairiang et al., 1992).

## Suggested Mechanisms of Carcinogenesis

These parasites, like other pathogens, secrete a lot of factors to modulate host environment in accordance with their specific needs. The whole genome sequencing has provided detailed insight into their life in bile duct and has identified several pathways that show that parasite is highly adapted to survive in lipid-rich diet from bile and cholangiocytes (Young et al., 2014). They also identified excretory secretory (ES) proteins in *O. viverrini* homologs to proteins capable of preventing interaction of LPS with TLR4. The association of these two parasitic infections with cancer has been studied in several experimental animal models, but these studies failed to provide direct evidence that infection with liver flukes alone is carcinogenic. So, three mechanisms have been proposed for CCA induction by *O. viverrini*: (1) feeding activities of parasite causes mechanical damage to biliary epithelia, (2) infection-related inflammation/mutations, and (3) toxic effect of parasite molecules, specially ES (Sripa et al., 2012a). In *O. viverrini* infection associated carcinoma, the epithelial lining of the cholangiocytes has been found to be associated with excretory/secretory proteins of *O. viverrini* (Sripa and Kaewkes, 2000; Pinlaor et al., 2009; Smout et al., 2009). The host cell internalizes excretory/secretory protein via clathrin caveolae endocytic pathway and induces inflammation by upregulating TLR4 on cholangiocytes that leads to release of IL6 and IL8 cytokines (Ninlawan et al., 2010). Therefore, ES proteins are suspected to be the antigens for CCA due to associated inflammation (Chaiyadet et al., 2015). In a study from Thailand, Sripa et al. (2012b) investigated the relationship between plasma levels of IL-6 and the risk of developing advanced fibrosis and bile duct cancer due to chronic *Opisthorchis* infection and found 58 times higher IL-6 concentration in plasma of individuals with advanced fibrosis compared with age, sex, and nearest-neighbor matched controls. They also noticed 221 times higher IL-6 concentration in individuals with bile duct cancer than controls. This chronic inflammation induces increase in oxidative stress through the formation of DNA damage lesions in the bile duct epithelial cells. Chan-On et al. (2013) used exosome sequencing to identify distinct mutational pattern between *O. viverrini*-infected and non-infected CCA among patients from Asia and Europe. The number of average somatic mutations burden per tumor was higher in infected CCA (26) compared with non-infected (16), suggesting non-liver fluke-mediated risk factors had lower mutagenic potential. Recently, Jusakul et al. (2017) performed a large-scale whole genome sequencing of 489 patients with CCA from 10 countries including fluke-positive and fluke-negative tumors. They identified distinct pathways of tumorigenesis in fluke-infected tumor, where it is initiated by genome-wide epigenetic derangement and subsequent spontaneous 5-methylcytosine deamination and CpG > TpG mutations. In contrast, in non-infected CCA, intrinsic genetic mutations are an initiating event followed by DNA methylation. Using the hamster model, researchers from Thailand (Pinlaor et al., 2004) performed a time course study of formation of 8-nitroguanine and 8-oxo-7,8-dihydro-2'-deoxyguanosine, iNOs expression and nitric oxide production, and their pathological effect. They demonstrated that repeated infections with *O. viverrini* leads to increased iNOS expression and proliferating cell nuclear antigen accumulation in the epithelial cells of bile duct from day 90 onward. This finding further supports the notion that inflammation-mediated DNA damage of the cells leads to CCA. In a recent animal model study, novel oxysterol-like metabolites were detected by liquid chromatography–mass spectroscopy in egg and adult parasite and same oxyseterol-like metabolites and DNA adduct were detected in bile of infected hamsters suggesting infection associated chromosomal lesions in host cells (Gouveia et al., 2017). They also noticed precancerous lesion conducive of malignancy like enlargement of liver, inflammation with severe periductal fibrosis, and biliary tract neoplasia by histology. Apart from direct effect of this parasite, it was also reported that these parasites increase susceptibility of host to secondary bacterial infections. In a study from Thailand, *O. viverrini* was found to be associated with *H. pylori* coinfection in patients with CCA (Deenonpoe et al., 2017).

## Cestodes and Nematodes

Several species of cestodes infect humans (*T. solium, T. saginata, T. asiatica, Hymenolepis*, etc.) and some of them like *Echinococcus* and *T. solium* can induce severe illness. Among cestodes, *Taenia* spp. make one of the most important class, as *T. solium* and *T. saginata* both have large endemic areas and infect several million people per year.

*T. solium* is a pork parasite; when infected pork carrying *Taenia* cysts is consumed (undercooked/raw), it causes taeniasis in humans (Prasad et al., 2008). Sometimes, these eggs either through contaminated vegetables or water find their way back to humans, and when ingested, they hatch and, through vascular routes, lodge into different parts of the body causing diseases like cysticercosis (skeletal muscles), ocular NCC (retina), or NCC (CNS). In CNS, it may remain innocuous for a prolonged period of time (maybe 3–4 years) without causing any apparent clinical symptoms. Later, in symptomatic phase, it causes seizures, which may be life-threating in rare cases. As in other helminthic infections, the cysts of parasite are associated with chronic inflammation at the site of infection or cyst lodging. Chronic inflammation in host tissues is a common feature of helminth infections and plays an important role in carcinogenesis induction by parasite. The longer the inflammation, the higher the risk of associated carcinogenesis, as immune cells (neutrophils, macrophages, eosinophils, etc.) generate large amounts of inflammatory cytokines, prostaglandins, and free radicals in the form of reactive oxygen and nitrogen species, which promote genetic instability and malignant transformation of cells. However, there are very few reports available suggesting the role of cestodes in carcinogenesis and that too involve *T. solium* studies only.

In an autopsy file-based study from Mexico, authors found more frequent NCC incidence in cases with malignant hematological diseases than in controls (*P* = 0.01) with odds ratio 3.54, thus strongly suggesting association of NCC and malignancy (Herrera et al., 1999). In another study, frequency of chromosomal aberration for chromosomes 7, 11, and 14 was analyzed using chromosome painting technique in lymphocytes from 10 NCC patients and control (Herrera et al., 2001). There was a significantly higher (*P* = 0.002) translocation frequency for these chromosomes in patients with NCC, where persistent *T. solium* antigen exposure that can cause chromosome instability was observed. Later, the same group reported an increased DNA damage in human lymphocyte when cultured with *T. solium* metacestode protein (Herrera et al., 2003). This finding shows strong association between *Taenia* infection and higher cancer risk. In another study from Brazil, effect of vesicular fluid and saline extract of *T. solium* metacestode was examined for its carcinogenic potential on *Drosophila melanogaster* wing somatic mutation and recombination test (SMART) (Silva et al., 2006). Both the test compounds were found genotoxic in both the cases. However, there is still no consensus in the scientific community regarding association of *Taenia* with cancer, and it needs to be further explored using new and advanced tools.

There are some reports of association of *Trichomonas crassiceps* with cancer occurrence, mainly in immunosuppressed individuals. However, when its role in cancer was evaluated in a mice model study after methyl-nitrosourea exposure, only 35% of infected mice developed lymphoma, in contrast with 50% of control non-infected animals (Ordoñez et al., 2003), thus ruling out any association. However, surprisingly, recently, there was a report of presence of this parasite in soft tissue tumor (Roesel et al., 2014) in an immunocompetent patient. As this infection happens only with immunosuppressed individual, this new report draws the need of a detailed study about its role in cancer with modern tools.

Similarly, not much is known about the role of nematodes in carcinogenesis. In one study, ES products from six species of nematodes were found to affect the cell proliferation of HT29-D4 and HGT-1 cell lines *in vitro* (Huby et al., 1995). ES products from *Trichostrongylus colubriformis* have been shown to induce cell proliferation in three epithelial intestinal cell lines (RIC, IEC-6, IRD-98) and in epithelial kidney cells (MDCK) (Huby et al., 1999). There are few case reports on the role of *Spirocerca lupi* in esophageal carcinoma of dogs (Banga et al., 2005). In contrast, infection or crude extract of *Trichinella spiralis* has been shown to have a protective role in carcinogenesis (Wang et al., 2009). Similarly, *E. granulosus* and *Fasciola hepatica* infection also has been associated with antitumor effect (Ferreira et al., 2018; Rostami Rad et al., 2018); however, the detailed mechanism is still illusive. Thus, the field is still wide open for further exploration before ascertaining their role in cancer.

## Conclusions and Perspectives

Despite mass deworming, the nuisance of intestinal infection is still a major public health concern, and its prevalence in some areas is increasing. There is a good chance that this mass deworming exercise will create drug-resistant strains, as only Praziquantel (PZQ)/Albandazole is used for this purpose, escalating the chance of helminth-associated chroming inflammation. It is estimated that chronic infection with biological agents (viral, bacterial, and parasitic) or other conditions with chronic inflammation contributes ~25% of all cancer cases worldwide. As with other cases, helminth-induced carcinogenesis is a complex process, which may involve several different mechanisms, but chronic inflammation is a key feature. This chronic inflammation may subsequently generate a microenvironment that might be conducive for the initiation and development of cancer. Association between parasitic infections and occurrence of cancers in humans is well-established only for some parasites. *S. haematobium, O. viverrini*, and *C. sinensis* are highly carcinogenic, while other species of the genera *Opisthorchis* (*O. felineus*) and *Schistosoma* (*S. japonicum*) have carcinogenic potential, but their role in carcinogenesis is not yet well-established. These helminths induce carcinogenesis in surrounding tissues primarily by three mechanisms, chronic inflammation, metabolic oxidative stress induced by parasitic products, and host tissue damage during parasite growth and development, along with the active wound healing. However, detailed understanding of host–parasite relationships, metabolic stress, effect of/on microbiome, functional consequences of parasitic, and host and environmental factors is still in nascent stage and requires a lot of attention from scientific community.

Immune metabolism is a new area that needs to be explored to understand immunopathogensis of diseases in a new light, this is especially important for helminthic diseases due to their strong immune-modulatory properties. Several studies have shown an inverse relationship between helminth infection and inflammatory disorders such as autoimmunity, allergies, diabetes, and inflammatory bowel disease (Aravindhan et al., 2010; Wiria et al., 2012). Wiria et al. (2013) found a negative association between risk factors (body mass index, waist-to-hip ratio, and lipid levels) of cardiovascular diseases and intestinal helminth infections. At the same time, metabolic changes/stress can lead to changes in immune cells' behavior and make them directed to chronic inflammation (Russell et al., 2019). Detailed study on this aspect of helminths is much needed.

Study of host manipulation by parasites had seen rapid growth with constant increasing interest of the scientific community, but the progress looks falter when we look for how parasite manipulate behavior (Herbison et al., 2018). Helminths not only promote inflammation, but they also have products that are antitumor in nature. Studies are going on to identify these products and their mechanisms. A detailed review of this aspect of helminth infection is described in the review by Callejas et al. (2018). Identifying factors that manipulate and associating them with gene expression shifts in parasite and changes in host will be the future direction for improvement of understanding. Another important aspect, which is generally overlooked in case of parasitic infection, is their effect on host microbiome. These helminths co-habitat the same niche as of microbiome of host, and the interactions between parasite and microbiome play important roles in outcome of infection. Of late, it is also evident that these parasites change the gut microbiome of host (Zhao et al., 2019), and altered gut microbiome is protective to the occurrence of gut-associated tumor or inflammatory disorder (Holzscheiter et al., 2014). Mice treated with antibiotics and anti-mycotics to deplete the host microbiome after *Schistosoma* infection had significantly less inflammation of intestine and decreased development of granuloma (Holzscheiter et al., 2014). In a recent study, among children from Zimbabwe treated with PZQ for *S. haematobium*, the diversity of gut microbiome was significantly different among infected and uninfected and was not related to PZQ treatment (Kay et al., 2015). These studies underline the importance of parasites and associated alteration in microbiota in their pathogenesis and serve as groundwork for future systematic studies to understand the intricate relationship of host and different helminths. A detailed review of biomarkers and role of microbiome is published by Scholte et al. (2018). In this review, the emphasis was on the need of a detailed study of phylogenetic and molecular evolutionary markers to understand the role of host parasite proteins and genes to identify new biomarkers.

Of late, it is well-established that these helminths make immune system suppressed or more inclined to Th2 response and promote secondary infection of other biological carcinogens (specially viruses). It will be interesting to have more knowledge of this relationship. Studies aiming for the identification of carcinogenic parasitic factors (metabolites, microRNAs, immune cell check point regulators, secretory proteins, etc.) and the related host cell mechanisms/signaling pathways that lead to tumorigenesis are much necessitated for proper understanding of this association. It will be very interesting to explore the role of non-coding RNAs, either secreted by parasite or synthesized by host cells, in cancer initiation (Arora et al., 2017). Detailed study of metabolic changes induced by these parasitic infections as well as metabolites synthesized by them that lead to cancer may give better target for therapeutic intervention of parasite-induced cancer. An improved understanding of these mechanistic pathways and identification of key molecular factors linking inflammation to cancer may ultimately provide molecular targets for prompt and early detection, prevention, and therapy of inflammation-associated cancers.

## Author Contributions

NA, RKa, ST, FA, RKu, AM, and AP deigned the manuscript. NA, RKa, ST, FA, RKu, and AP wrote the manuscript. NA, AM, and AP drafted and critically evaluated the manuscript.

### Conflict of Interest

The authors declare that the research was conducted in the absence of any commercial or financial relationships that could be construed as a potential conflict of interest.
